# The Ultrastructural Localization of Type II, IV, and VI Collagens at the Vitreoretinal Interface

**DOI:** 10.1371/journal.pone.0134325

**Published:** 2015-07-31

**Authors:** Shao Chong Bu, Roel Kuijer, Roelofje J. van der Worp, Xiao Rong Li, Johanna M. M. Hooymans, Leonoor I. Los

**Affiliations:** 1 Department of Ophthalmology, University Medical Center Groningen, University of Groningen, Groningen, the Netherlands; 2 Department of Biomedical Engineering, FB40, University of Groningen, University Medical Center Groningen, A. Deusinglaan 1, building 3215, FB40, 9713 AV, Groningen, The Netherlands; 3 W.J. Kolff Institute, Graduate School of Medical Sciences, University of Groningen, Groningen, the Netherlands; 4 Tianjin Medical University Eye Hospital, Tianjin Medical University, Tianjin, China, Fu Kang Street 251, Nan Kai District, Tianjin, 300384, China; National Center for Scientific Research Demokritos, GREECE

## Abstract

**Background:**

The vitreoretinal interface is the border of the cortical vitreous and the inner surface of the retina. The adhesion of the cortical vitreous to the ILM, namely vitreoretinal adhesion, involves a series of complex molecular adhesion mechanisms and has been considered as an important pathogenic factor in many vitreoretinal diseases. The presence of type VI collagen at the vitreoretinal interface and its possible interaction with collagens and glycoproteins indicates that type VI collagen may contribute to the vitreoretinal adhesion.

**Purpose:**

To clarify the ultrastructural location of type VI collagen and its relationship to type II and IV collagens at the vitreoretinal interface.

**Methods:**

The ultrastructural localization of type II, IV and VI collagens in the adult human vitreoretinal interface of five donor eyes was evaluated by transmission electron microscopy using immunogold labeling.

**Results:**

In the pre-equatorial region, we observed densely packed vitreous lamellae with a partly intraretinal course containing type II and VI collagens, reticular structures containing type IV and VI collagens and a thin inner limiting membrane (ILM) containing type IV and VI collagens in a linear distribution pattern. From the anterior to the posterior retina, the linear pattern of type IV and VI collagen labeling gradually became more diffusely present throughout the entire thickness of the ILM.

**Conclusions:**

The presence of type VI collagen in vitreous lamellae penetrating the ILM into the superficial retina suggests that type VI collagen may be involved in the organization of vitreous fibers into lamellae and in the adhesion of the vitreous fibers to the retina. The close relation of type VI to type IV collagen in the ILM suggests that type VI collagen is an important collagen type in the ILM. The topographic variations of type IV and VI collagens in the different regions of the ILM suggest a regional heterogeneity of the ILM. The reticular labeling pattern of type IV and VI collagens observed in the anterior vitreous are highly similar to labeling patterns of blood vessel walls. In the anterior vitreous, they may represent remnants of the regressed embryonic hyaloid blood vessel system. Their presence is in support of the theory on interactive remodeling of the developing vitreous as opposed to the main stream theory of displacement and compression of the primary by the secondary vitreous.

## Introduction

The vitreoretinal interface is the border of the cortical vitreous and the inner surface of the retina. It is a complex extracellular matrix (ECM) structure containing cortical vitreous, retinal inner limiting membrane (ILM) and Müller cell endfeet. The major ECM components of the vitreoretinal interface are collagens, glycosaminoglycans (GAGs) and glycoproteins (GPs). Cortical vitreous consists of densely packed heterotypic fibrils containing type II, V/XI and IX collagens. While type II collagen forms the main scaffold of the vitreous body, the other ECM proteins, including type IX collagen, GAGs and GPs, are responsible for stabilizing the collagen network. The chondroitin sulphate chains of type IX collagen and opticin on the surface of the type II collagen fibrils probably maintain the space between the collagen fibrils and prevent their self-aggregation. Hyaluronan is highly hydrated and fills the spaces between the collagen fibrils [1].

The ILM is essentially the basement membrane of retinal Müller cells and it consists of basement membrane associated ECM proteins of which type IV collagen, laminin, fibronectin and others have been identified [2, 3]. The assembly of the basement membrane is initiated by deposition of laminins on the cell surface and interactions between laminins and cell surface receptors (such as the integrin family and dystroglycan) [4, 5]. Type IV collagen forms a network as the main scaffold of the basement membrane which interacts with the laminin network through specific molecular mediators, such as nidogen/entactin and type VI collagen [6–8]. The scaffold formed by the interdependent laminin and type IV collagen networks thus provides sites for other basement membrane associated molecules to bind and to interact with the adjacent stromal tissue.

The adhesion of the cortical vitreous to the ILM, namely vitreoretinal adhesion, involves a series of complex molecular adhesion mechanisms and has been considered as an important pathogenic factor in many vitreoretinal diseases [9, 10]. Previous research indicated that regional differences in vitreoretinal attachment mechanisms may exist. At the vitreous base, the cortical vitreous fibrils run perpendicularly to the retina and penetrate through the ILM of the retina forming a sub-ILM network structure and thus mediating a strong adhesion with the underlying tissue [11, 12]. With age, the width of the vitreous base increases and its posterior edge gradually extends more posteriorly [13]. At the pre-equatorial and equatorial regions, areas were found where vitreous fibrils focally penetrate the ILM and where ILM-associated type IV collagen was seen to be focally interrupted at sites where it extends itself into the vitreous cortex [12]. Based on previous clinical and histological studies, the presence of focal attachments is also probable at the posterior pole, and specifically in areas overlying retinal blood vessels, the macula and optic disc. Kishi et al reported that cortical vitreous remnants were found on the macular surface of the retina in 22% (26 out of 59) of autopsy eyes that had had spontaneous posterior vitreous detachment (PVD) [14]. At the posterior pole, cortical vitreous fibers run parallel to the retinal surface, and there is only very limited evidence showing that the vitreous fibrils may penetrate the ILM forming adhesions with the underlying retina. Gandorfer et al found a direct insertion of native type II vitreous collagen fibrils into the collagenous network of the ILM at the rim of idiopathic macular holes [15]. Focal vitreoretinal attachments may explain why—in case of a spontaneous PVD—retinal tissue may be damaged. Thereby, intravitreal hemorrhage, retinal tear formation, and ultimately rhegmatogenous retinal detachment may occur.

Overall and focal vitreoretinal attachment have been studied previously, but only limited information on the specific collagens involved is available. Previous research has indicated that non-collagenous molecules, such as fibronectin, laminin, and opticin are involved in the vitreoretinal adhesion at the posterior pole. These molecules may interact with both type II collagen in the vitreous and type IV collagen in the ILM and help to connect the vitreous to the ILM [3, 16, 17]. Kohno et al reported a linear and laminar distribution of fibronectin and laminin in the equatorial and posterior ILM and suggested that fibronectin and laminin are responsible for the vitreoretinal adhesion because of their ability to interact with various extracellular components in the vitreous fibrils and the ILM [3]. A possible involvement of opticin in vitreoretinal adhesion was proposed because of its presence on the surface of vitreous collagen fibrils and its capacity to bind to heparan sulphate proteoglycans at the inner portion of the ILM. This could not be confirmed in an opticin knockout mouse model, because of the absence of a spontaneous PVD [17]. These findings indicate that other adhesion mechanisms contribute to vitreoretinal attachment.

The incomplete knowledge concerning the molecules involved in vitreoretinal adhesion, contributes to making current treatment options for vitreoretinal adhesion related diseases limited and inefficient. Techniques to create a PVD should ensure that a complete PVD is achieved, in order to avoid increased focal vitreoretinal traction, which may exacerbate pre-existing conditions. This can be done surgically, but also pharmacologically. Non-collagenous adhesion molecules have been specifically targeted by injecting enzymes such as chondroitinase, dispase, hyaluronidase and recombinant plasmin into the vitreous [18–21]. Pre-clinical studies suggested that such enzymes can release vitreoretinal adhesion to a certain extent. However, clinical studies found limited effectiveness and serious side effects in some cases [22]. At present, Ocriplasmin, the truncated form of plasmin containing a specific proteolytic activity against fibronectin and laminin, is commercially available for clinical use. It may induce complete posterior vitreous detachment in human and rabbit eyes. However, a randomized clinical trial for the treatment of vitreomacular traction syndrome reported only limited effectiveness. It reported vitreoretinal separation 28 days after Ocriplasmin injection in 26.5% of cases versus 10% in placebo treated eyes [23]. In another case series, the release of vitreomacular adhesion occurred in 47.1% of the treated eyes (8/17 eyes) [21]. This limited effectiveness of Ocriplasmin indicates that additional adhesion molecules may be involved in vitreoretinal adhesion.

In previous studies, a number of collagens including type II, V, VI, IX and XI in the vitreous and type IV, VI, VII and XVIII in the ILM have been identified [24, 25]. Type VI collagen is an anchoring fibril which can interact with various ECM components such as type I, II, IV and XIV collagens, fibronectin, perlecan, biglycan, decorin and hyaluronan [8, 26–32]. The presence of type VI collagen at the vitreoretinal interface and its possible interaction with type II and IV collagen, hyaluronan and fibronectin indicates that type VI collagen may contribute to the vitreoretinal adhesion. However, there are conflicting results concerning the exact location and distribution of type VI collagen in the vitreoretinal interface. Bishop et al identified type VI collagen fibrils in bovine and human vitreous by their morphological features using rotary shadowing electron microscopy and immunoblotting results confirmed the presence of type VI collagen in bovine vitreous [25]. However, morphological evaluation alone might be considered incomplete evidence and immunoblotting did not confirm the presence of type VI collagen in human vitreous [25]. Ponsioen et al reported the presence of type VI collagen in human ILM but did not find type VI collagen in the cortical vitreous by using immunohistochemical staining and light microscopic (LM) evaluation [24].

In the present study, by using immuno-transmission electron microscopy (TEM), we compared the distribution patterns of type II, IV and VI collagens in the anterior (pre-equatorial), equatorial and posterior regions of the vitreoretinal interface. Hereby, we hope to contribute to the understanding of vitreoretinal adhesion at different anatomic locations.

## Materials and Methods

### 2.1 Technovit 8100 embedding

Five human eyes of 5 donors (35, 56, 66 and 77 years old male, and 78 years old female) without any known ophthalmic disorders were obtained from the Euro Cornea Bank (Beverwijk, the Netherlands. (http://www.eurotissuebank.nl/comeabank/) after removal of the cornea for corneal transplantation. In the Netherlands, the usage of donor material is provided for by a law named “Wet op Orgaan Donatie (WOD)”. Following this law, donors provide written informed consent for donation with an opt out for the usage of left-over material for related scientific research. Specific requirements for the usage for scientific research of left-over material originating from corneal grafting have been described in an additional document formulated by the Ministry of Health, Welfare, and Sport and the BIS foundation (Eurotransplant; Leiden, July 21, 1995; 6714.ht). The current research was performed in accordance with all requirements stated in the WOD and the concerning document. Approval of the local medical ethics committee was not required as the data were analyzed anonymously.

The tissue processing and the immuno-TEM procedures were adopted from the protocols of Ponsioen et. al. with some modifications [12]. Briefly, the donor eyes (without cornea) were fixed by immersion in 2% paraformaldehyde (PF) for 1 hour. Two penetrating sclerotomies about 5 mm in diameter were made at the equatorial region to facilitate the infiltration of the fixatives and the embedding reagents. Then, the eyes were fixed in 2% PF for another 4 hours and washed in 6.8% sucrose in phosphate buffered saline (PBS) for 16 hours. After brief washing in distilled water, the eyes were dehydrated in gradient acetones (30 to 100%) and embedded in Technovit 8100 (T8100; Heraeus Kulzer, Wehrheim, Germany). After infiltration with T8100A (without accelerator) at 4°C, the eyes were transferred to −20°C for infiltration with T8100A+B (with accelerator), and again to 4°C for polymerisation.

The embedded eyes were cut and sections of 3 μm thickness were stained with toluidin blue (TB) for evaluation by LM. The pre-equatorial area, the equator and the posterior pole were selected for morphological and immunohistochemical evaluation by TEM. Sections with a thickness of approximately 200 nm were mounted on formvar-coated nickel grids, subjected to the immuno-TEM procedures described below, and evaluated by a Philips 201 TEM (Amsterdam, the Netherlands) operated at 80 kV.

### 2.2 Immunogold labeling for type II, IV and VI collagens

The 200 nm sections were pretreated with 0.1% trypsin in Tris-HCL (pH 7.8 containing 0.1% CaCl_2_) for 15 minutes at 37°C. Then, the sections were washed in PBS and incubated in 0.1 M citric acid (pH 3.0) for 30 minutes at 37°C. After washing in PBS, the sections were incubated in PBS with 0.15% glycine, 5% bovine serum albumin (BSA), 2% rabbit serum (for anti-type VI collagen antibody) or 2% goat serum (for anti-type II and IV collagen antibodies) for 30 minutes at room temperature to block the non-specific binding of the primary antibodies. Afterwards, the sections were incubated in the primary antibodies diluted in PBS (1/100) with 1% BSA-c (Aurion, Wageningen, the Netherlands) first for 2 hours at 37°C, and then overnight at room temperature. The primary antibodies included: goat anti-type II collagen (polyclonal, Southern Biotechnology Associates (SBA), Birmingham, USA), goat anti-type IV collagen (polyclonal, SBA) and rabbit anti-type VI collagen (polyclonal, Abcam, Cambridge, UK). The next day, the sections were washed in PBS and incubated in 6 nm gold particles conjugated to secondary antibodies (Rabbit anti Goat IgG or Goat anti-Rabbit IgG; Aurion) diluted in PBS (1/150) for 60 minutes at room temperature. Then, the sections were washed in PBS, incubated in 2% glutaraldehyde in PBS for 2 minutes, and briefly washed in double distilled water. A silver enhancement solution (Aurion R-gent enhancer, Aurion) was then applied for 10 minutes at room temperature. After washing in double distilled water, the sections were counterstained with uranyl acetate in 25 cP Methyl cellulose (MC-UAc, Sigma-Aldrich, St. Louis, USA). The negative controls underwent the entire procedure, except that the primary antibodies were omitted.

### 2.3 Double-labeling of type IV and VI collagens using sequential immunogold labeling with silver enhancement

To obtain differently sized immunogold labeling to type IV and VI collagens, a three-step immuno-histochemical procedure was used which resulted in the attachment of 15 nm gold particles to type IV collagen, and a two-step procedure with silver enhancement was used to label the type VI collagen with larger particles. Briefly, the 200 nm sections went through the same antigen retrieval processes as aforementioned. The sections were incubated in a mixture of diluted goat anti-type IV collagen and rabbit anti-type VI collagen antibodies (1/100) in PBS with 1% BSA-c (Aurion) at 4°C overnight. On the second day, the sections were washed in PBS and incubated in mouse anti-goat IgG antibody diluted in PBS containing 1% BSA-c (Aurion) for 30 minutes. After washing in PBS, the sections were incubated in 6 nm gold particles conjugated to goat anti-rabbit IgG antibody (Aurion) diluted in PBS containing 1% BSA-c (Aurion) at room temperature for 45 minutes. Afterwards, the sections were washed in double distilled water and incubated in silver enhancement kit (Aurion) for 10 minutes at room temperature. This procedure resulted in the immunogold labeling of type VI collagen with silver enhancement, thus creating relatively large electron dense particles. Subsequently, the sections were washed in double distilled water and incubated in 15 nm gold particles conjugated to goat anti-mouse IgG antibody diluted in PBS containing 1% BSA-c (Aurion) at room temperature for 45 minutes. Thus, type IV collagen was labeled by the 15 nm gold particles. After washing in double distilled water, the sections were counterstained with uranyl acetate in 25 cP MC-UAc at 4°C for 15 minutes.

## Results

### 3.1 General observations

The ILM contains densely packed filaments forming an interwoven network. The filamentous network configuration of the ILM was most prominent at the pre-equatorial and equatorial areas. At the posterior region, the ILM is thickened and indented at its retinal surface. The vitreous surface of the ILM does not have an entirely smooth aspect, but it has numerous branching filaments extending themselves towards the vitreous body.

**Fig 1:**
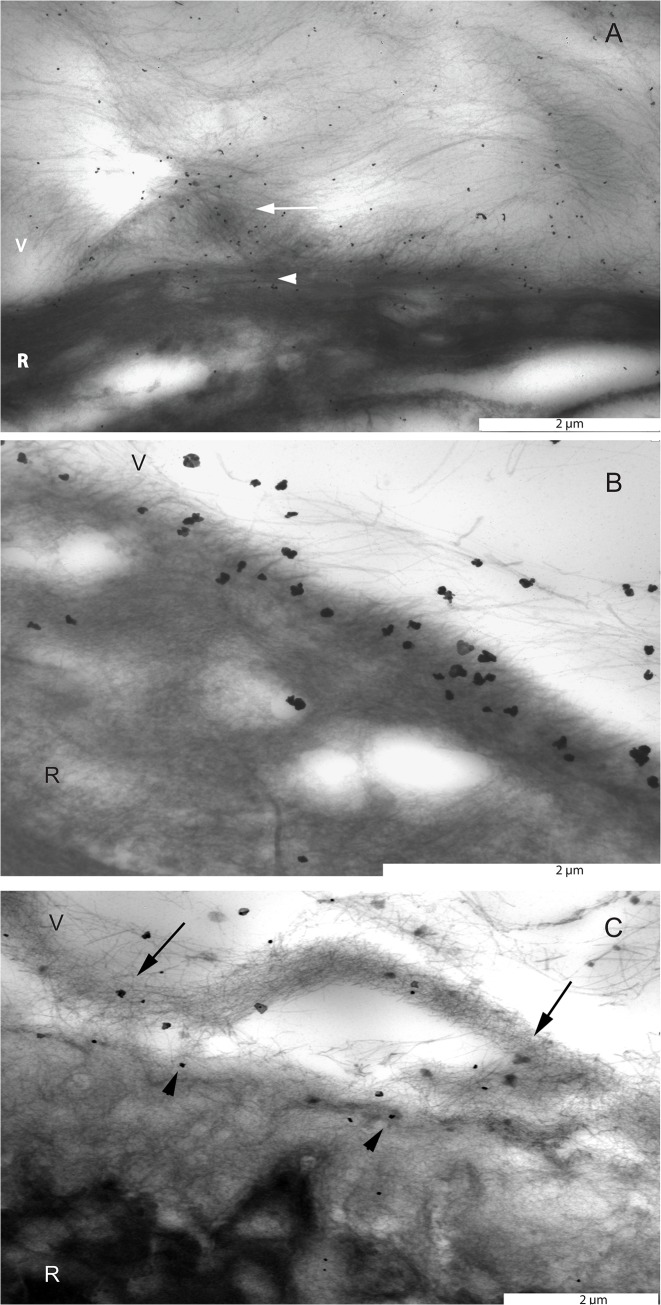
Transmission electron microscopic images of the pre-equatorial, equatorial and posterior vitreoretinal interface stained with an antibody against type II collagen. A. Eye of a 56 year-old donor. Immunogold labeling directed at type II collagen was located on individual vitreous fibers in the basal vitreous and superficial retina. Type II collagen positive fibers in the basal vitreous were often seen to condense and form lamellar structures (arrow). Vitreous fibers penetrated the ILM, which is indicative of attachment of these fibers to the superficial retina (arrow head). B. Eye of a 35 year-old donor. Vitreous fibers containing immunogold labeling to type II collagen at the equator. Note that gold labelled fibers penetrate the ILM and attach themselves to the superficial retina. C. Eye of a 66 year-old donor. At the posterior vitreoretinal interface, immunogold labeling directed at type II collagen was located on the cortical vitreous fibers (arrows) and the vitreal side of the inner limiting membrane (arrow heads). Bar = 2μm. V = vitreous; R = retina.

**Fig 2:**
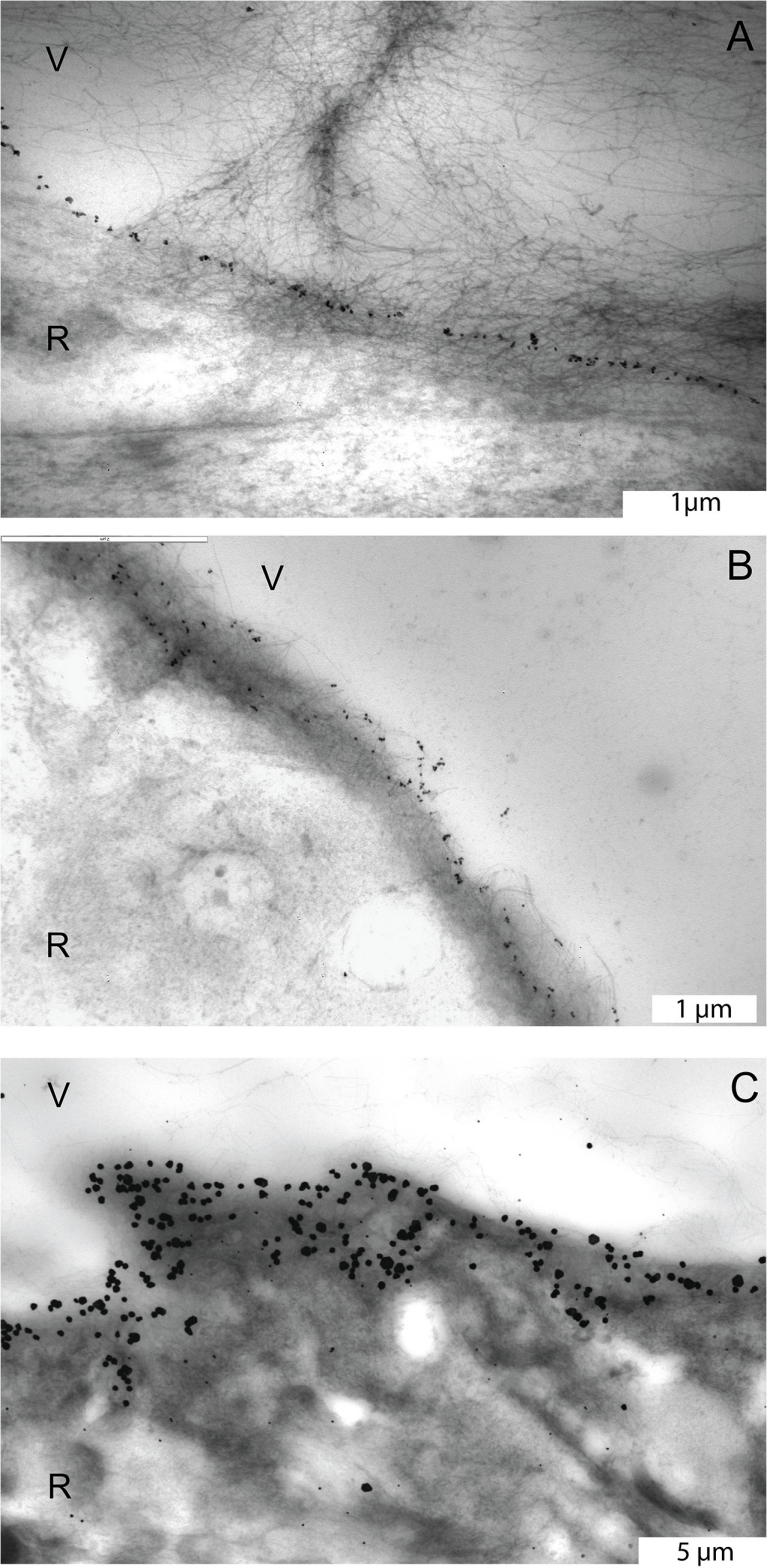
Transmission electron microscopic images of the pre-equatorial, equatorial and posterior ILM stained with an antibody against type IV collagen. A. Pre-equatorial ILM of the eye of the 56 year-old donor. Note the linear distribution pattern of the labeling. B. Equatorial ILM of the eye of the 77 year-old donor. Note the more diffuse staining pattern. C. Posterior vitreoretinal interface of the 56 year-old donor eye. Type IV collagen appears to be distributed throughout the entire thickness of the ILM. A and B, bar = 1 μm; C, bar = 5 μm. V = vitreous; R = retina.

**Fig 3:**
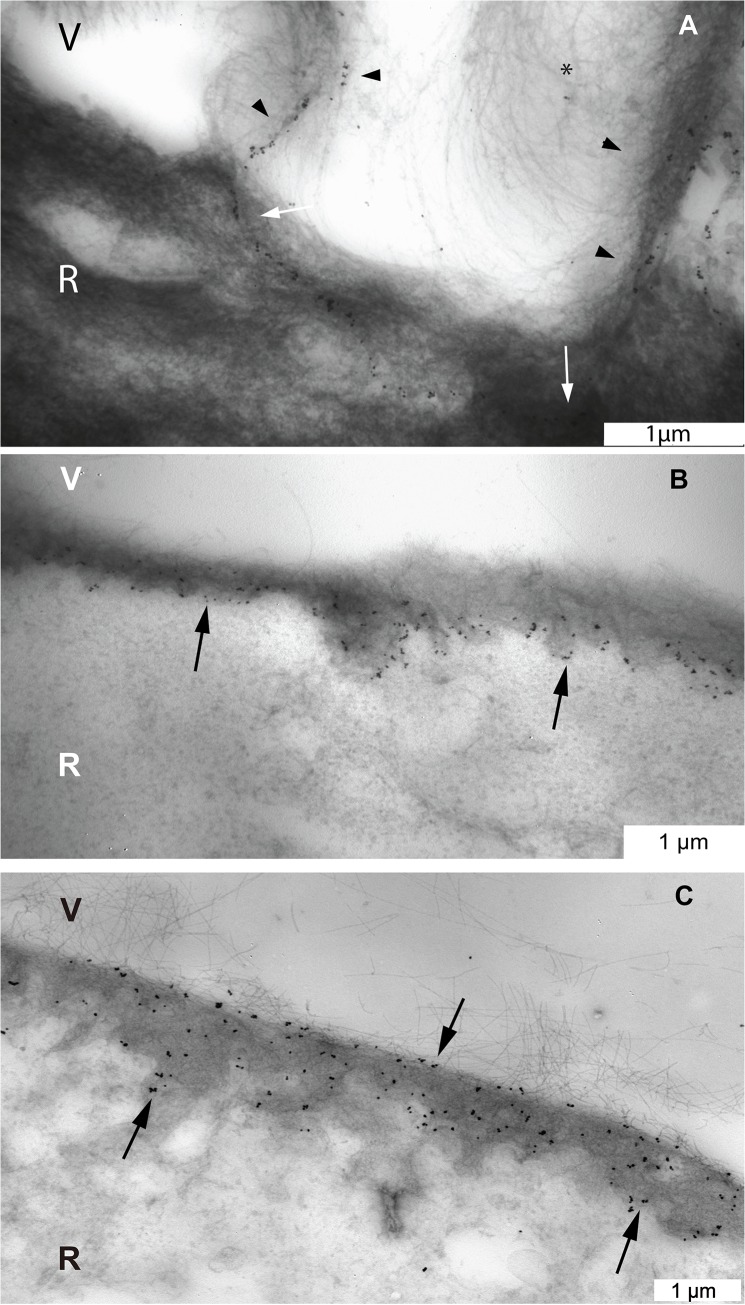
Transmission electron microscopic images of the vitreoretinal interface stained with an antibody against type VI collagen. A. In the eye of the 56 year-old donor, type VI collagen labeling was found on the vitreous lamellae that run perpendicular to the pre-equatorial retina (black arrow heads), on fine cortical vitreous fibers (asterisks) and in the superficial retina (white arrows). B. In the eye of the 77 year-old donor, type VI collagen was observed at the equatorial ILM in a thicker than just linear band (black arrows). C. In the eye of the 56 year-old donor, type VI collagen was distributed throughout the entire thickness of the posterior ILM (black arrows). V = vitreous; R = retina. Bar = 1 μm.

**Fig 4:**
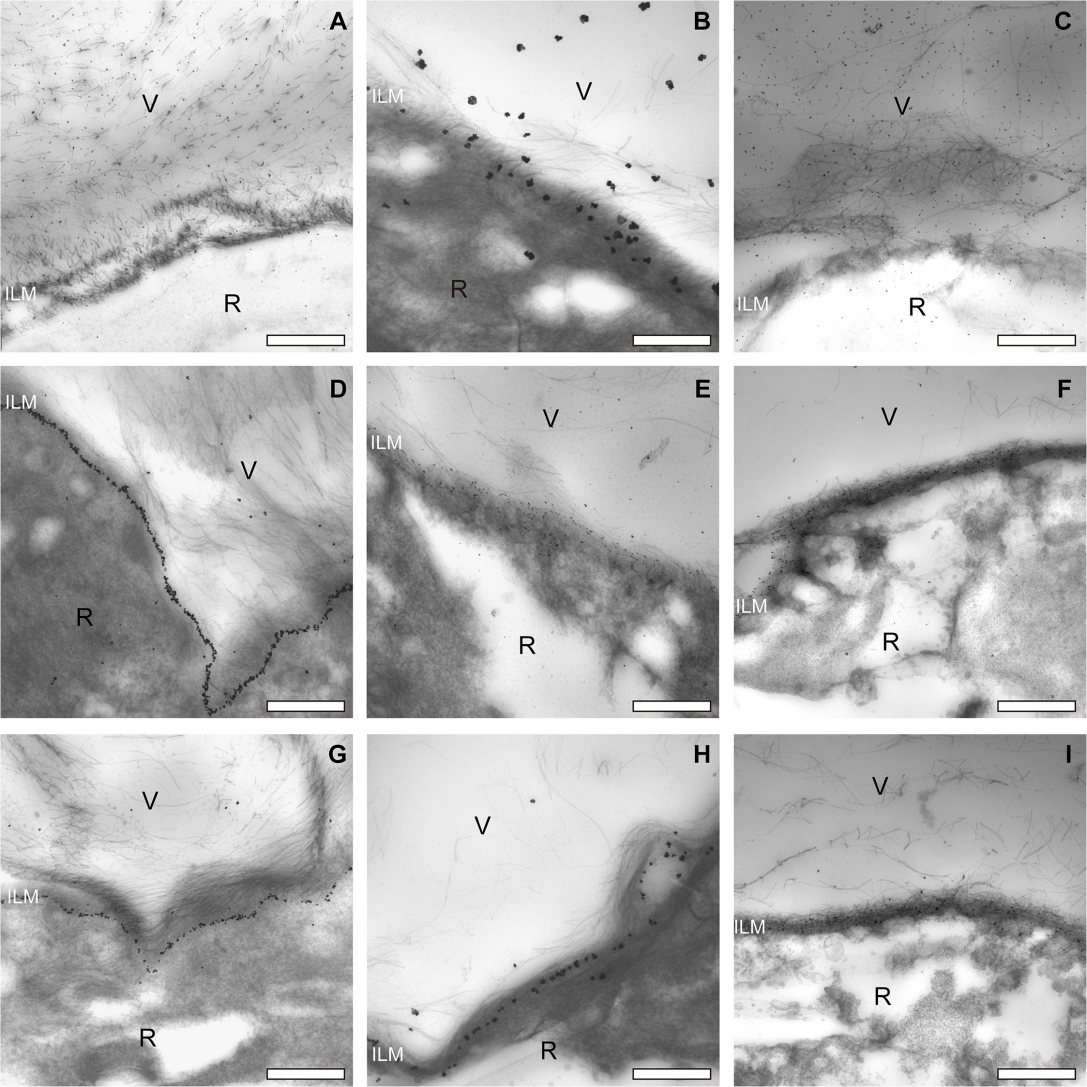
Complete set of transmission electron microscopic images of the pre-equatorial, equatorial and posterior vitreoretinal interface of one donor (male, aged 35 years). Type II collagen staining of vitreous fibrils at the pre-equatorial (A), equatorial (B) and posterior (C) vitreoretinal interface. Type IV collagen staining of the ILM at the pre-equatorial (D), equatorial (E) and posterior (F) vitreoretinal interface. Note the lineair labeling pattern at the pre-equatorial location, and the more diffuse distribution of the labeling at the equator and posterior sites. Type VI collagen staining of the ILM and cortical vitreous at the pre-equatorial (G), equatorial (H) and posterior (I) vitreoretinal interface. The ILM thickens towards the posterior retina. In this younger donor, the ILM at the posterior pole is thinner than that in the older donor (compare with Fig 5). Bar = 2μm. V = vitreous; R = retina; ILM = inner limiting membrane.

**Fig 5:**
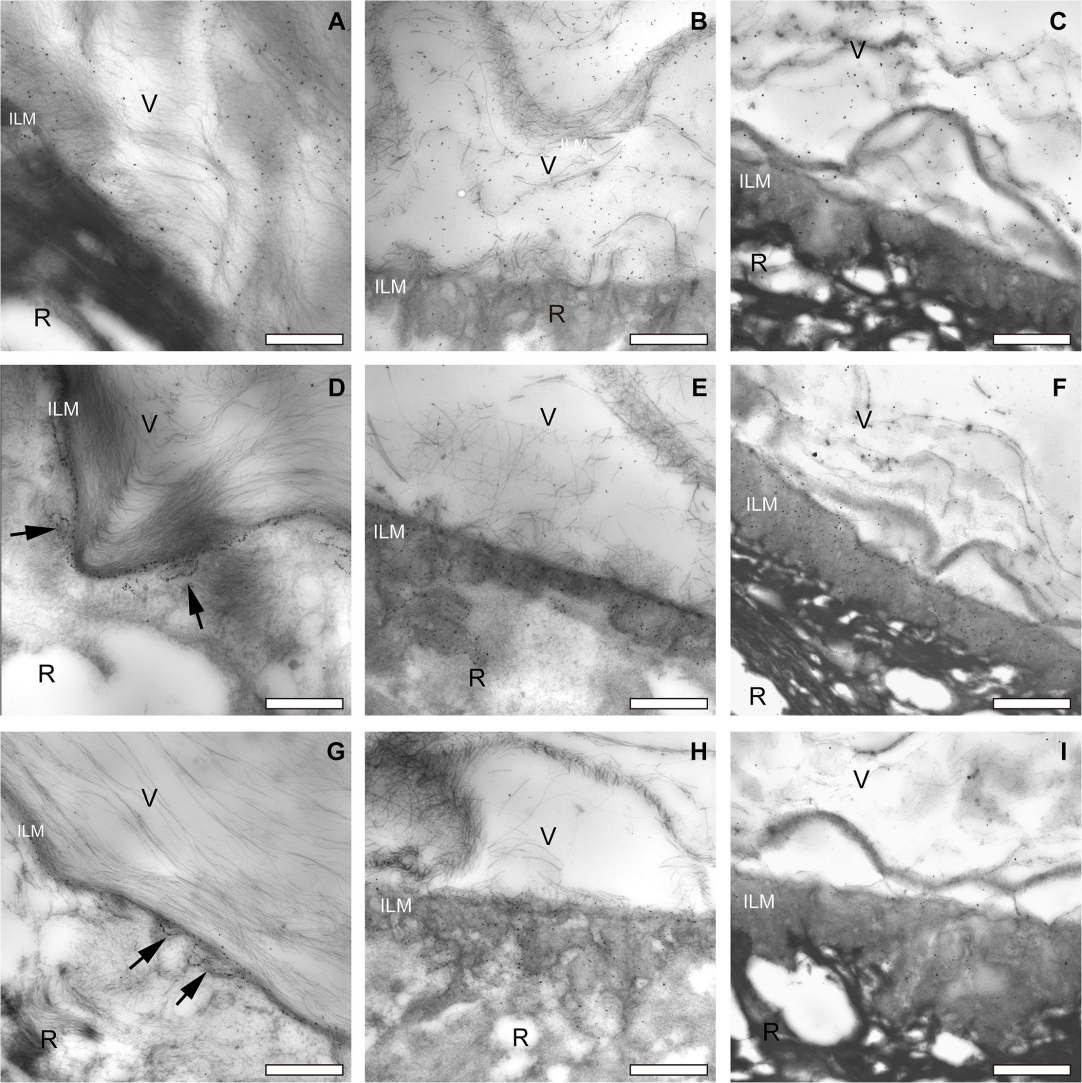
Complete set of transmission electron microscopic images of the pre-equatorial, equatorial and posterior vitreoretinal interface of one donor (male, aged 66 years). Type II collagen staining of vitreous fibrils at the pre-equatorial (A), equatorial (B) and posterior (C) vitreoretinal interface. Type IV collagen staining of the ILM at the pre-equatorial (D), equatorial (E) and posterior (F) vitreoretinal interface. Type VI collagen staining in the pre-equatorial (G), equatorial (H) and posterior (I) vitreoretinal interface. Note the lineair labeling pattern at the pre-equatorial location, and the more diffuse distribution of the labeling at the equator and posterior sites. D and G: note reticular pattern (arrows). Also, the ILM thickens towards the posterior retina, in this older donor, the ILM at the posterior pole is thicker than that in the younger donor (compare with Fig 4). Bar = 2μm. V = vitreous; R = retina; ILM = inner limiting membrane.

**Fig 6:**
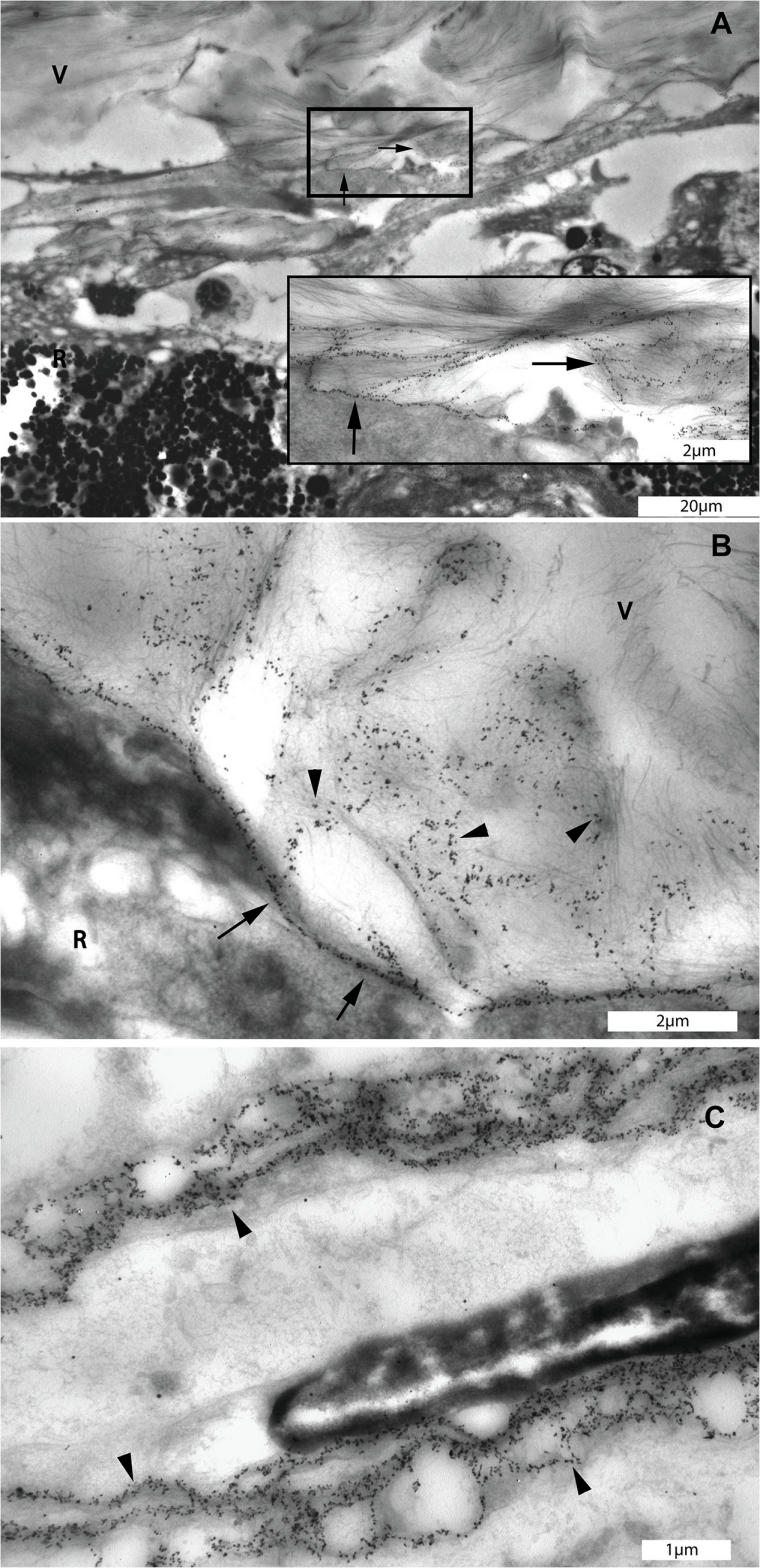
Transmission electron microscopic images of diffuse networks which were stained with type IV collagen antibody at the pre-equatorial vitreo-retinal interface of the eye of the 56 year-old donor. A. Immunogold labeling of type IV collagen displayed a reticular pattern at the pre-equatorial vitreoretinal interface (square box). Insert: Detail of marked area in A: Type IV collagen positive structures in the vitreous extending themselves to the adjacent retina (black arrows). B: Linear distribution of type IV collagen indicating the location of the ILM (arrows) and type IV collagen positive structures in the vitreous attaching themselves to the ILM and superficial retina (black arrow heads). C. Distribution of type IV collagen in the basement membrane of a choroidal blood vessel displaying a similar reticular pattern as that found in the pre-equatorial intravitreal reticular structures (black arrow heads). A, bar = 20 μm, insert, bar = 2 μm; B, bar = 2 μm; C, bar = 1 μm. V = vitreous; R = retina.

**Fig 7:**
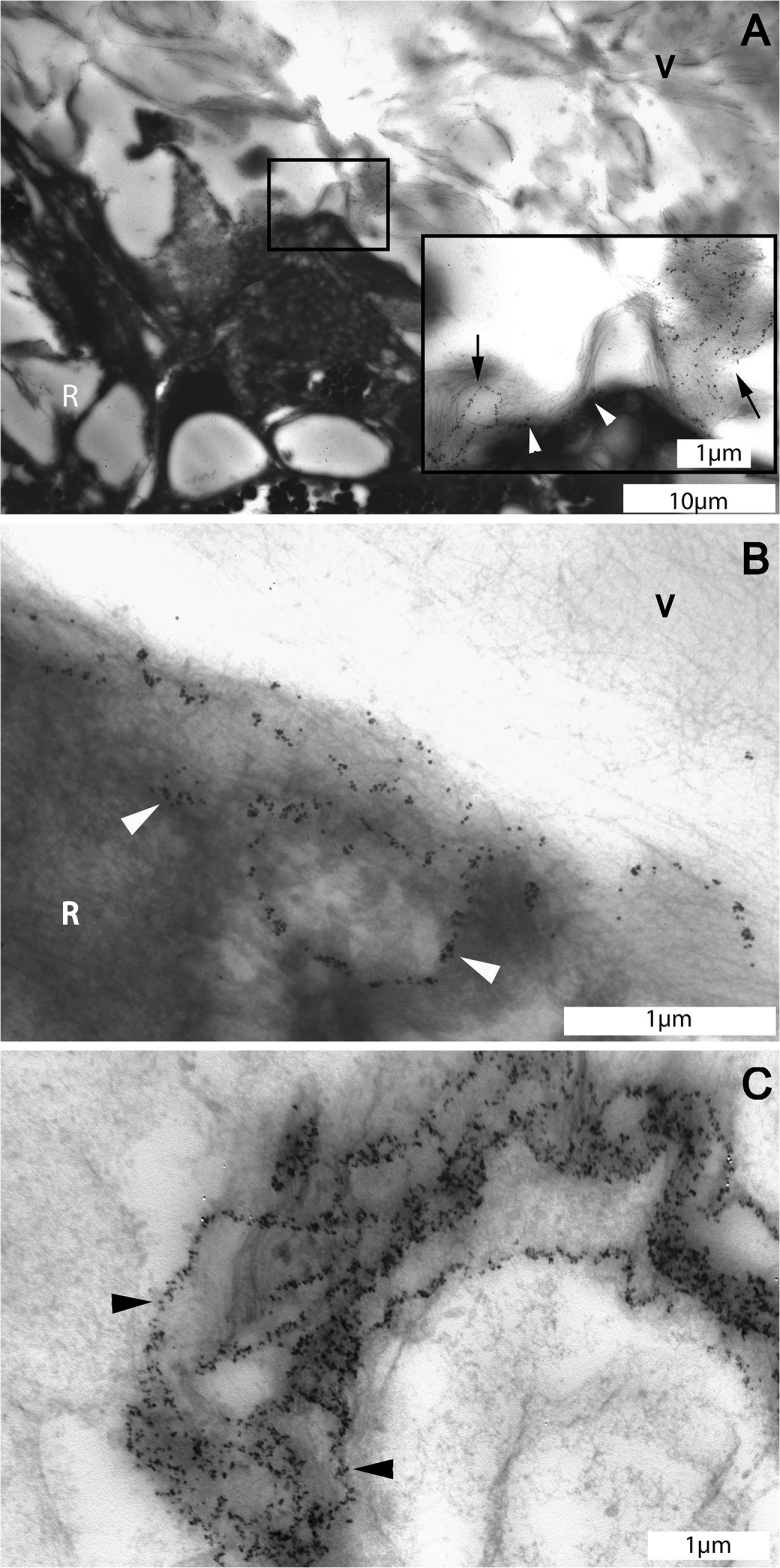
Transmission electron microscopic images of the pre-equatorial vitreoretinal interface of the eye of the 56 year-old donor. A. Overview of the pre-equatorial vitreoretinal interface stained for type VI collagen. Insert: Detail ofmarked area in A: Type VI collagen forms reticular structures in the vitreous (arrows) that extend themselves towards the superficial retina (arrow heads). B: Type VI collagen forms a reticular pattern in the superficial retina at the pre-equatorial area (arrow heads). C. Type VI collagen in the basement membrane of a retinal blood vessel displayed a pattern similar to that found in the pre-equatorial reticular intravitreal structure (arrow heads). A, bar = 10 μm, insert, bar = 1 μm; B and C, bar = 1 μm. V = vitreous; R = retina.

1. Pre-equatorial area: The majority of vitreous fibers are condensed into lamellae. Vitreous fibers attach themselves to the ILM but they also penetrate the ILM and attach themselves to the superficial retina. At the pre-equatorial area, the ILM is thin and it has an electron density that is comparable to that of the retina, which makes it difficult to clearly delineate the two tissues (Figs 1–7).
2. Equatorial area: At the equatorial ILM, vitreous fibers were observed in the 35 and 66 years old donors (Figs 1B, 4B, 4E, 4H, 5B, 5E and 5H), but not in the 77 years old donor (Figs 2B and 3B). In all evaluated eyes, the ILM gradually thickened (from the anterior to the posterior part of the eye) and indentations at its retinal side were observed (Figs 1–5).
3. Posterior pole: The ILM was thicker in this area compared to the pre-equatorial and equatorial regions. Furthermore, retinal indentations of the ILM became more prominent both in number and extent (Figs 1–5). At the equator and posterior pole, the electron density of the ILM was higher than that of the subjacent retina, thus making it possible to delineate the two tissues (Figs 1–5).

### 3.2 The ultrastructral localization of type II, IV and VI collagen in vitreoretinal interface

The overall localization of type II, IV and VI collagens in the vitreoretinal interface was summarized in Table 1. Additionally, overall collagen distribution patterns were consistent between the younger (35 years old, Fig 4) and the older donor (66 years old, Fig 5).

**Table 1:** Summary of the collagens present at the vitreoretinal interface.

	Pre-equatorial area			Equator			Posterior pole		
	Vitreous	ILM	Inner retina	Vitreous	ILM	Inner retina	Vitreous	ILM	Inner retina
Type II collagen	(+)	(-)	(+)	(+)	(-)	(+)	(+)	(-)	(+)
Type IV collagen	(+)*	(+)	(-)	(-)	(+)	(-)	(-)	(+)	(-)
Type VI collagen	(+)	(+)	(+)	(+)	(+)	(-)	(+)	(+)	(-)

ILM: inner limiting membrane;

*type IV collagen is attached to reticular structures in the anterior vitreous.

#### 3.2.1. Type II collagen

Immunogold labeling directed at type II collagen was identified on the vitreous fibers. At the pre-equatorial region of all donor eyes, the vitreous was attached to the retina and vitreous lamellae and individual vitreous fibers were found to penetrate the ILM and to attach themselves to the underlying retina. Gold labeling was observed in a substantial part of the superficial retina (Figs 1, 4 and 5). In the 35 and 66 years old donors, individual vitreous fibers were seen to penetrate the ILM at the equator and posterior pole and to attach themselves to the ILM or the superficial retina (Figs 4 and 5). In the 66 years old donor, the posterior cortical vitreous was partially detached from the superficial retina (Fig 5).

#### 3.2.2. Type IV collagen

At the pre-equatorial and equatorial areas, immunogold labeling directed at type IV collagen displayed a linear configuration (Figs 2A, 4D and 5D). From the equatorial area towards the posterior pole, labeling was seen to gradually become more diffusely spread throughout the entire thickness of the ILM (Figs 2B, 2C, 4E, 4F, 5E and 5F).

#### 3.2.3. Type VI collagen

At the pre-equatorial area, immunogold labeling directed at type VI collagen was diffusely distributed throughout the cortical vitreous and was frequently identified to be concentrated in a linear pattern alongside the densely packed vitreous lamellae which attached themselves to the basal retina (Fig 3A). The linear labeling continued intraretinally and was seen to be oriented parallel to the surface of the ILM, at variable depths in relation to the retina (Fig 3A). This would be consistent with a penetration of vitreous lamellae into and their attachment to large parts of the superficial retina. Further towards the posterior pole, the linear labeling pattern was lost and the labeling was seen to be diffusely distributed over the entire thickness of the ILM (Figs 3B, 3C, 4H, 4I, 5H and 5I).

### 3.3 Reticular labeling patterns

Reticular structures containing both type IV and VI collagens were identified at the pre-equatorial region of the vitreoretinal interface. These reticular structures were located intra-vitreally, were attached to the ILM and extended themselves into the superficial retina. The labeling pattern was highly similar to that found in basement membranes of retinal and choroidal blood vessels (Figs 5D, 5G, 6 and 7).

### 3.4 Double labeling of type IV and VI collagens

**Fig 8:**
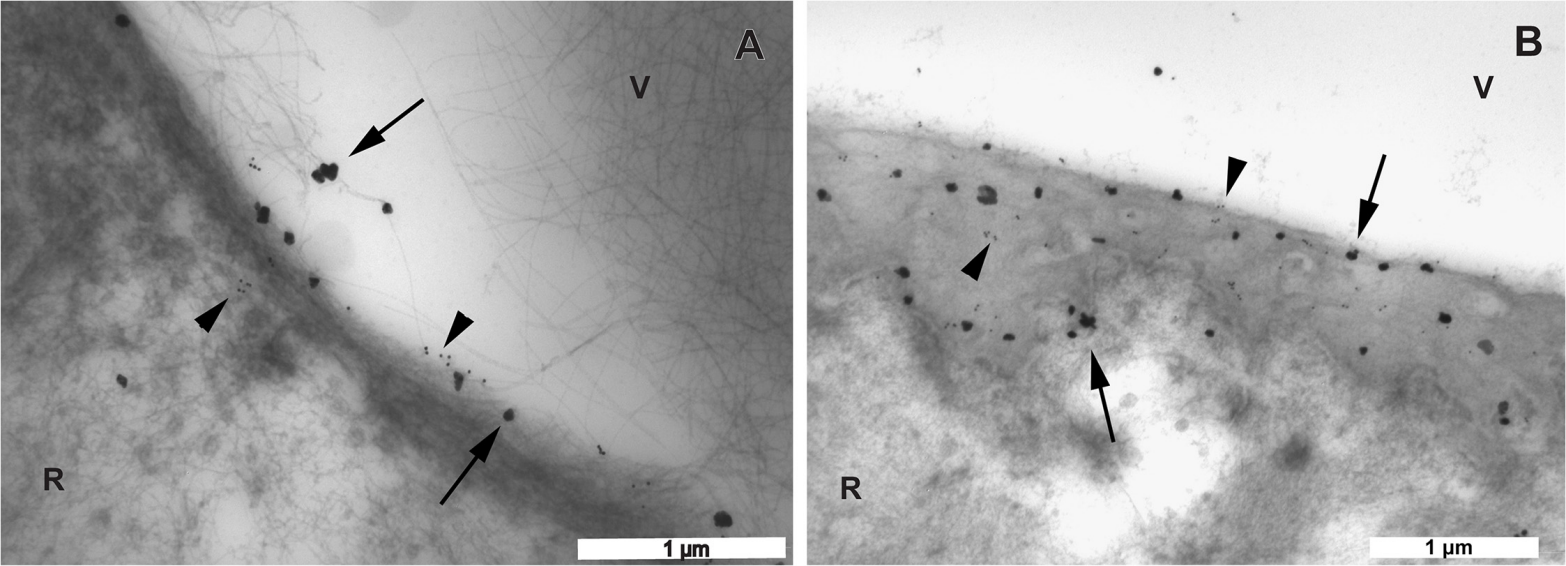
Transmission electron microscopic images of the vitreoretinal interface double labeled with antibodies against type IV and VI collagens. The large labels are gold particles with silver enhancement representing type VI collagen (arrows). The small labels are 15 nm gold particles representing type IV collagen (arrow heads). A. In the eye of the 56 year-old donor, the equatorial area exhibits a linear distribution pattern of both type IV and VI collagens. Note that type VI collagen can also be seen on the vitreous fibrils. B. In the eye of the 35 year-old donor, towards the posterior pole, both type IV and VI collagens become diffusely distributed over the entire thickness of the ILM. A, B bar = 1 μm. V = vitreous; R = retina.

The double labeling of type IV and VI collagens displayed a similar distribution pattern: a linear labeling pattern at the pre-equatorial ILM, and a gradually more diffuse distribution of labeling in the direction of the posterior ILM. Additionally, an intense staining in the basement membranes of retinal and choroidal blood vessels was observed. Furthermore, the 6 nm gold label with silver enhancement directed to type VI collagen could be detected on the vitreous fibrils (Fig 8).

## Discussion

The current immuno-TEM study expands on previous studies by providing detailed information on the site-specific ultrastructural distribution of type II, IV and VI collagens at the vitreoretinal interface. These types of collagen are not restricted to either the vitreous or the retina, but encompass a wider area at the vitreoretinal interface. Thus, they are likely involved in vitreoretinal attachment. Type IV and VI collagens have similar distribution patterns at the ILM, whereas type VI collagens can also be found diffusely in the vitreous cortex and located to intravitreal lamellae. Type IV and VI collagens stain reticular-like structures at the vitreous base. These labeling patterns are highly similar to those found in retinal and choroidal blood vessel walls, which indicates a vascular origin of these structures.

### 4.1 Pre-equatorial region

At the pre-equatorial vitreoretinal interface, we observed densely packed vitreous lamellae with an intraretinal course containing type II and VI collagens, reticular-like structures containing type IV and VI collagens that span a wide vitreoretinal area and a thin ILM containing type IV and VI collagens in a linear distribution pattern.

In previous studies, type VI collagen was mainly found in the connective tissue closely associated with basement membranes and it was proposed to function as an anchoring fiber [33]. Because of its presence in vitreous lamellae and superficial retina, it may thus be involved in organizing vitreous fibers into lamellae and in anchoring the intraretinal vitreous fibers to the surrounding matrix and superficial retinal cells. The basal vitreoretinal adhesion has been well documented by LM and TEM studies. The adhesion mechanism includes the interruption of the thin ILM that allows the vitreous collagen fibers to attach themselves to the underlying fibrils in the superficial retina and crypts between the Müller cells [11, 34]. The direct binding ability of type VI collagen to various ECM components could promote the anchoring of vitreous fibers to the retina. Furthermore, by interacting with integrin α1β1 and α2β1, type II and VI collagens may form attachments with the ILM and retinal Müller cells, which could be one of the adhesion mechanisms of the vitreous fibers and superficial retina [35–37]. The ultrastructural location and staining pattern of type VI collagen in the pre-equatorial vitreous indicates an anchoring function of type VI collagen by a direct interaction with type II and IV collagens in the vitreoretinal interface. In skin, type VI collagen forms a flexible network that intertwines with collagen fibers and anchors them to the surrounding connective tissue [38]. Furthermore, previous studies using immunogold-TEM co-localized type VI and IV collagens on the endothelial side of the glomerular basement membrane and in the basement membrane of the placenta [39, 40]. The specific direct interaction of type IV and VI collagens demonstrated by Kuo et al indicates that type VI collagens anchor the basement membrane to the surrounding matrix through non-covalent bindings [8]. Therefore, the presence of type VI collagen in the cortical vitreous lamellae adjacent to the ILM supports our hypothesis that type VI collagen is involved in vitreoretinal adhesion by mediating the interactions of vitreous fibers to the surrounding ECM fibrils and cells. A possible anchoring function of type VI collagen in case of an epiretinal membrane was previously described [41] and is illustrated in S1 File, S1 and S2 Figs.

Type IV and VI collagens are known components of the basement membrane of blood vessels [42–44]. In the present study, we confirmed their presence in retinal and choroidal blood vessels. Strikingly, the labeling pattern of these collagens in the blood vessel wall closely resembled that of the reticular labeling pattern observed at the pre-equatorial vitreoretinal interface. Since the adult vitreous body is avascular, we hypothesize that the stained intravitreal structures may represent remnants of the hyaloid vascular system. This would be in line with previous studies in the rabbit vitreous, where type IV collagen containing vascular remnants of the hyaloid system were found to be present throughout the adult rabbit vitreous and to be associated with intravitreal lamellae [45, 46].

The residual vascular remnants found at the pre-equatorial vitreoretinal interface support the theory that the secondary vitreous is formed by a process of interactive remodeling of the primary vitreous [47, 48]. The vascular remnants in the pre-equatorial vitreous indicate that the secondary vitreous is interwoven with the primary vitreous which contains the hyaloid vascular system.

This hypothesis on the development of the secondary vitreous, is in conflict with the main stream theory. The main stream theory proposed that the avascular–secondary–vitreous body starts to form between the primary vitreous and retina, and surrounds and compresses the primary vitreous. While the secondary vitreous gradually increases its volume, the primary vitreous ceases to grow and regresses. Based on this theory, the vascular remnants would be located exclusively in the center of the vitreous. This theory is supported by the observation of large vessel remnants in the center of the vitreous sometimes found by slit lamp microscopy and optical coherence tomography [49, 50]. The rest of the hyaloid vascular system, except for the central arteries, is assumed to disappear without leaving a trace. The alternative theory of the development of secondary vitreous was originally proposed by Jokl and Pau [51, 52]. They suggested that the retracting hyaloid vascular system acts as a scaffold for the secondary vitreous to organize itself, and that the formation of the secondary vitreous is the result of continuous remodeling of the primary vitreous. This theory was later supported by the finding of vascular remnants associated with intravitreal lamellae of the secondary vitreous in adult rabbit vitreous [46, 53]. Our immuno-TEM observations further expand on these previous findings by showing possible residual vascular remnants at the adult human vitreoretinal interface.

### 4.2 Regional variations in the ILM

Previous studies already found a regional variability in morphological aspects of the ILM. These observations were confirmed in the present study and consisted of a thin ILM anteriorly without indentations, and thickening of the ILM towards the posterior pole with the development of indentations at its retinal side. Also, anteriorly, the electron density of the ILM was comparable to that of the subjacent retina, whereas the ILM towards the posterior part of the eye has a higher electron density than the subjacent retina. The present study expands on existing knowledge by clarifying the site-specific arrangement of type IV and VI collagens at the retinal ILM. The linear labeling pattern of type IV and VI collagens at the pre-equatorial ILM changes into a more diffuse labeling of the entire thickness of the ILM towards the posterior pole. This suggests a regional heterogeneity of this basement membrane. Such heterogeneity would be consistent with previous publications on regional differences in collagenous composition of basement membranes in organs such as kidney and lung [39, 54]. Desjardins and Bendayan proposed that the heterogeneity in the composition of the renal basement membranes may be related to regional differences in function [55].

Type IV collagen is the predominant ECM protein in human ILM which accounts for about 57% of the total proteins [56]. Several studies indicated that type IV collagen is critical not only for the structural integrity of the basement membrane but also for neuron survival and angiogenesis [57, 58]. The type IV collagen isoform α5(IV)α5(IV)α6(IV) is the predominant heterotrimer in adult human ILM [59, 60]. Mutations in the COL4A5 and COL4A6 genes which compromise the production of α5(IV)α5(IV)α6(IV) heterotrimers, result in thinning of the ILM and nerve fiber layer in Alport’s syndrome [60, 61]. Our study showed a diffuse distribution of type IV collagen throughout the entire thickness of the posterior ILM, which might explain that the posterior retina is most susceptible to alterations in the composition of type IV collagen, and thus is seen to display significant pathological alterations in this genetic disease [60].

Previous studies showed that type VI collagen forms a fine beaded filament network in the vicinity of basement membranes, whereas the expression of type VI collagen in the basement membrane was only reported in the renal glomerular basement membrane and intestinal epithelial basement membrane [39, 62]. Our study also shows the presence of type VI collagen in the retinal ILM. Furthermore, the double-labeling of type IV and VI collagens in the ILM demonstrated a similar distribution pattern, which indicates that type VI collagen may interact with the type IV collagen network at this site. The proposed function of type VI collagen in these basement membranes is to regulate the synthesis and organization of fibronectin. Sabatelli et al reported an alteration of fibronectin organization in the ECM of type VI collagen-deficient fibroblasts [29]. Groulx et al reported that depletion of type VI collagen induced an upregulation of fibronectin expression and deposition resulting in a significant increase in cell spreading and fibrillar adhesion formation of intestinal epithelial cells [62].

The presence of type IV and VI collagens throughout the entire thickness of the posterior ILM, suggests that these collagens are synthesized during the aging process. In our series as well as in previous studies, the ILM has been shown to gradually thicken towards the posterior pole with increased numbers of ILM indentations at its retinal side. These extensions of the ILM, as suggested by previous authors, are considered to be age-related depositions of ECM onto the retinal aspect of the ILM [56]. Additionally, an age-related thickening of the ILM has been described by several reports [2, 11, 56]. Therefore, type IV and VI collagens located in these structures, are likely produced during the postnatal period. As for the origin of these collagens, we speculate that retinal Müller cells are candidate producers.

It has been suggested that the majority of the ILM proteins such as type IV collagen and laminin, are produced by lens and ciliary body epithelium and are then transported through the vitreous during the embryonic period [2]. After birth, the production of ECM proteins in these tissues is dramatically decreased. Previous work of our group showed that retinal Müller cells, the predominant glia in the retina, are possibly involved in the dynamic ECM turnover at the vitreoretinal interface [63]. In vitro, Müller cells can produce a broad spectrum of collagens including type IV and VI collagens and in vivo their endfeet are attached to the retinal side of the ILM [64]. Hyalocytes, the resident cells in the cortical vitreous, can also produce ECM proteins in vitro. However, it is not known whether these cells can produce type IV and VI collagens. Also, in contrast to Müller cells, hyalocytes are very scarce, and their presence is restricted to limited areas within the vitreous cortex [65]. Therefore, we suggest that type IV and VI collagens in the extensions of the ILM likely originate from retinal Müller cells.

The immuno-TEM technique allows the target protein to be localized with high precision. This technique has found wide application in studying the ultrastructure of the basement membrane and its adjacent ECM. However, the procedure has certain limitations which should be taken into account. One of the important issues is the specificity of the antibodies. In this study, we used commercially available antibodies and applied a double labeling technique to type IV and VI collagens to reduce the chance of false interpretations due to antibody cross-reaction. We observed type IV and VI collagen labeling at different sites within the ILM and the vascular basement membrane. In addition, type VI collagen labeling was observed on vitreous fibers and lamellae, whereas type IV collagen labeling on these structures was absent. Combined with the results of the negative controls that ran parallel with each immuno-TEM procedure, it is likely that the immunogold labeling directed at type II, IV and VI collagens is specific.

## Conclusions

In conclusion, our findings concerning the ultrastructural distribution of type II, IV and VI collagens indicate that the distribution of collagens in the ILM is heterogeneous. Type IV and VI collagens may form a single layer network in the pre-equatorial ILM and encompass the entire thickness of the ILM towards the posterior pole. Immunogold labeling for type IV collagen was exclusively found in the basement membrane and on the reticular structures in the anterior vitreous, whereas type VI collagen was additionally observed on vitreous lamellae and fibrils closely associated with the ILM. These findings indicate that type VI collagen could function as an anchoring fibril to organize vitreous fibers into lamellae and to attach them to the ILM and the underlying retina. The reticular labeling patterns of type IV and VI collagens support the hypothesis of interactive remodeling of the vitreous during its embryonic development.

## Supporting Information

S1 File Fibrocellular proliferation containing type IV and VI collagen in vitreoretinal interface. At the retina of a 77 year-old donor without any known ophthalmic disorders, a fibrocellular proliferation (epiretinal membrane, ERM) containing type IV (S1 Fig) and VI (S2 Fig) collagens was identified on the vitreal side of the posterior retina by immuno-gold transmission electron microscopy. (The eye was obtained from the Euro Cornea Bank and processed as described in the Methods section). Type VI collagen fibers were observed in areas of focal adhesion between ERM and ILM, which indicates that type VI collagen may be involved in mediating these adhesions.
(DOCX)

S1 Fig Transmission electron microscopic image of the posterior vitreoretinal interface of a 77 years old donor eye with an epiretinal membrane (ERM) stained with an antibody against type VI collagen. The ERM containing type VI collagen positive fibers showed focal attachments to the ILM (arrow heads). ERM = epiretinal membrane; ILM = inner limiting membrane. Bar = 1μm.
(TIF)

S2 Fig Transmission electron microscopic image of the posterior vitreoretinal interface of a 77 years old donor eye with an epiretinal membrane (ERM) stained with an antibody against type IV collagen. The fibrocellular membrane lies on the vitreal side of the inner limiting membrane (ILM), which is positive to the antibody against type IV collagen. The type IV collagen staining displayed a linear pattern in the ERM (arrows) and a diffuse pattern in the ILM. ILM = inner limiting membrane; EC = epiretinal cell; V = vitreous; R = retina. Bar = 2μm.
(TIF)
