# Supplementary material for: The Ultrastructural Localization of Type II, IV, and VI Collagens at the Vitreoretinal Interface
Source: PLoS One. 2015 Jul 31;10(7):e0134325. doi: 10.1371/journal.pone.0134325 (PMC4521792; doi:10.1371/journal.pone.0134325)
Supplement: S1 File — At the retina of a 77 year-old donor without any known ophthalmic disorders, a fibrocellular proliferation (epiretinal membrane, ERM) containing type IV (S1 Fig) and VI (S2 Fig) collagens was identified on the vitreal side of the posterior retina by immuno-gold transmission electron microscopy. (The eye was obtained from the Euro Cornea Bank and processed as described in the Methods section). Type VI collagen fibers were observed in areas of focal adhesion between ERM and ILM, which indicates that type VI collagen may be involved in mediating these adhesions. (DOCX) [file pone.0134325.s001.docx]

S1 File. Fibrocellular proliferation containing type IV and VI collagen in vitreoretinal interface

At the retina of a 77 year-old donor without any known ophthalmic disorders, a fibrocellular proliferation (epiretinal membrane, ERM) containing type IV (S1 Fig.) and VI (S2 Fig.) collagens was identified on the vitreal side of the posterior retina by immuno-gold transmission electron microscopy. (The eye was obtained from the Euro Cornea Bank and processed as described in the Methods section). Type VI collagen fibers were observed in areas of focal adhesion between ERM and ILM, which indicates that type VI collagen may be involved in mediating these adhesions.
