# Supplementary figures and images for: The Ultrastructural Localization of Type II, IV, and VI Collagens at the Vitreoretinal Interface
Source: PLoS One. 2015 Jul 31;10(7):e0134325. doi: 10.1371/journal.pone.0134325 (PMC4521792; doi:10.1371/journal.pone.0134325)

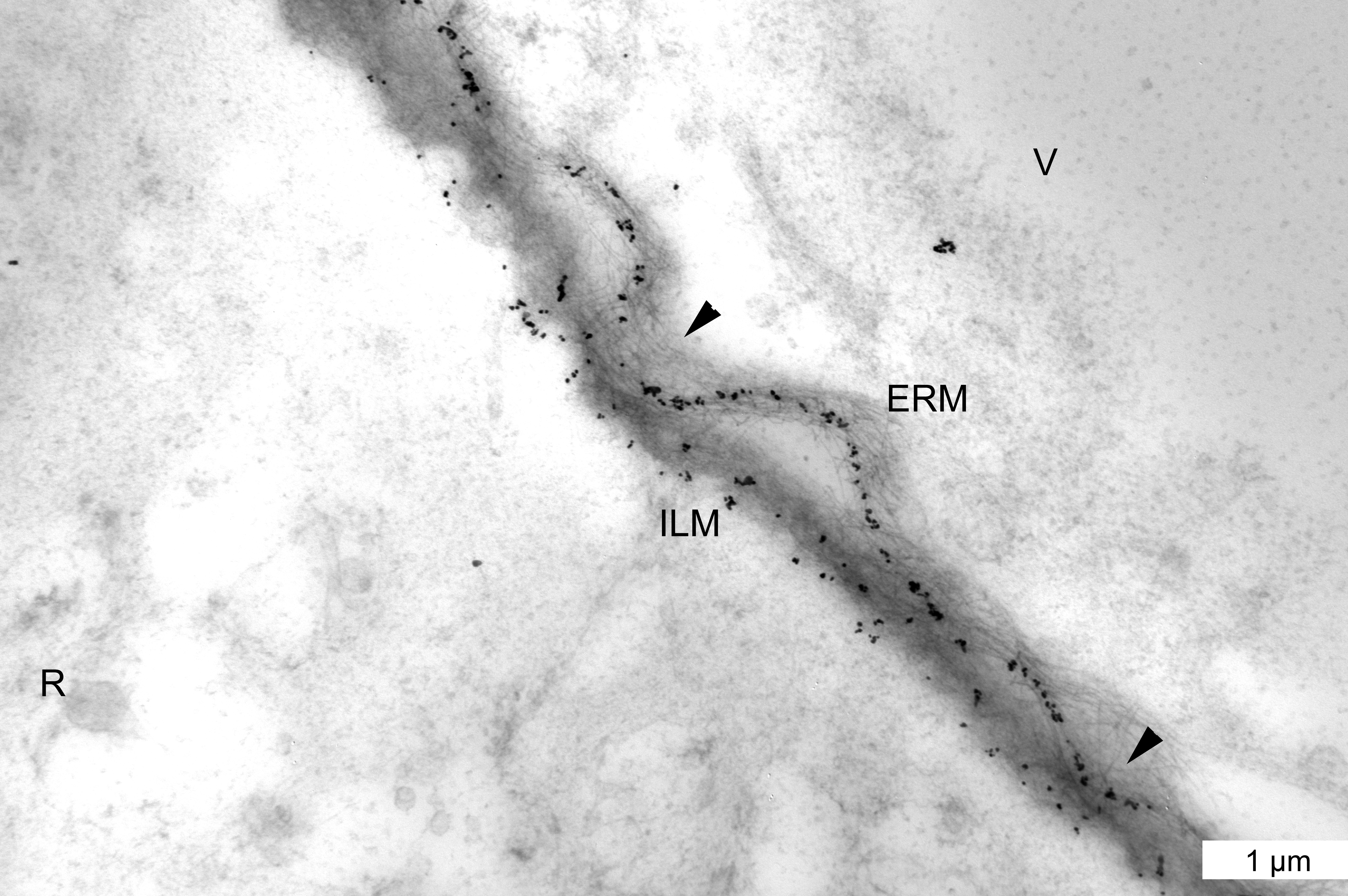

Supplement: S1 Fig — The ERM containing type VI collagen positive fibers showed focal attachments to the ILM (arrow heads). ERM = epiretinal membrane; ILM = inner limiting membrane. Bar = 1μm. (TIF) [file pone.0134325.s002.tif]

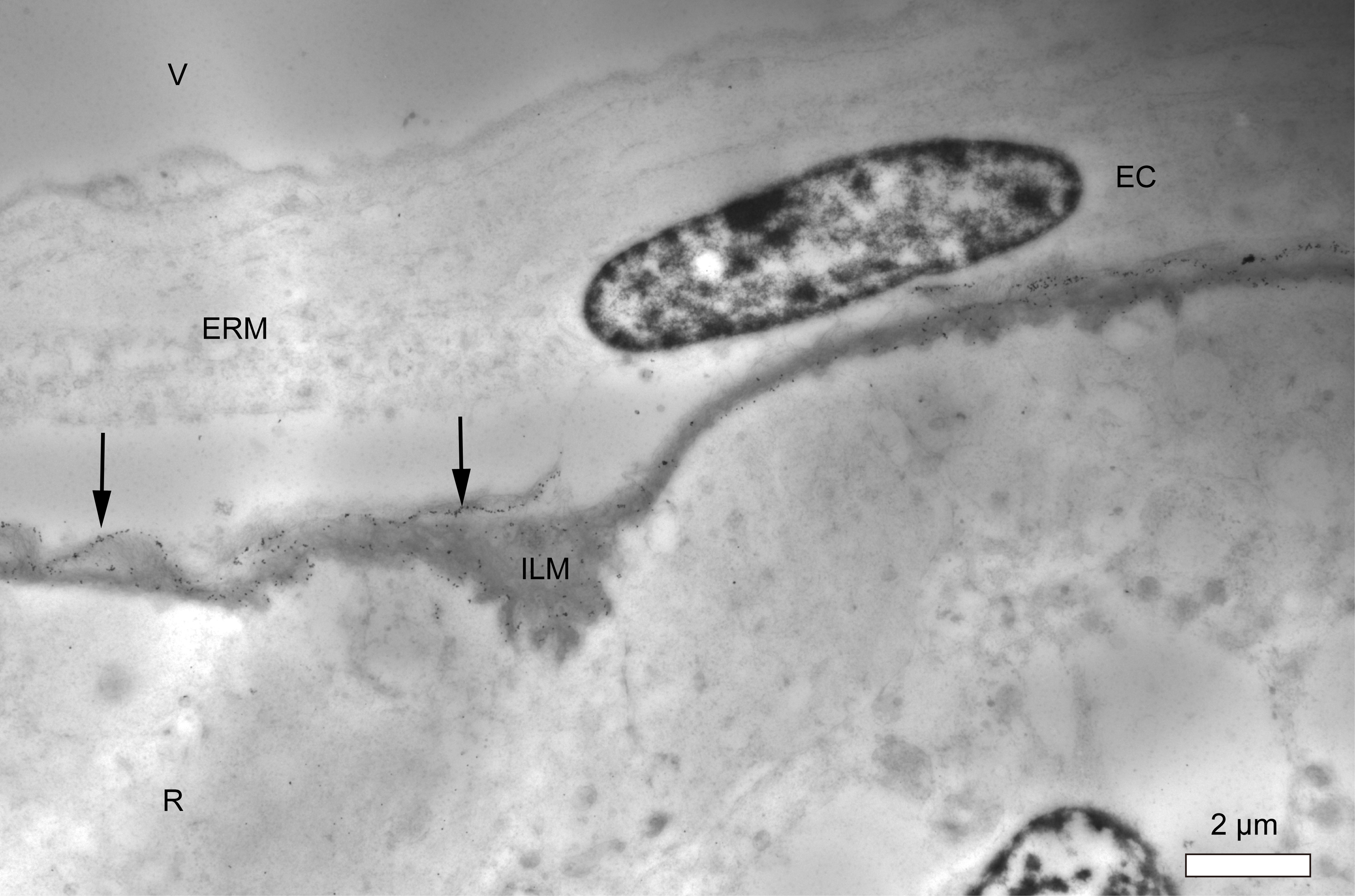

Supplement: S2 Fig — The fibrocellular membrane lies on the vitreal side of the inner limiting membrane (ILM), which is positive to the antibody against type IV collagen. The type IV collagen staining displayed a linear pattern in the ERM (arrows) and a diffuse pattern in the ILM. ILM = inner limiting membrane; EC = epiretinal cell; V = vitreous; R = retina. Bar = 2μm. (TIF) [file pone.0134325.s003.tif]
